# Association between infertility prevalence and exposure to heat stress: an ecological study based on the Universal Thermal Climate Index

**DOI:** 10.3389/fpubh.2026.1948152

**Published:** 2026-10-16

**Authors:** Dian Wang, Jiahua Qian, Xiaoming Zhang, Chunbao Mo, Yihao Chen, Yuhe Si, Yicheng Wang, Jianan He, Jianxiong Ma

**Affiliations:** 1 Department of Nephrology, The First Affiliated Hospital of Zhejiang Chinese Medical University (Zhejiang Provincial Hospital of Chinese Medicine), Hangzhou, Zhejiang, China; 2 Department of Public Health, The Reproduction Hospital of Guangxi Zhuang Autonomous Region, Nanning, Guangxi, China; 3 Guangxi Key Laboratory of Artificial Intelligence for Genetic Diseases of Long-dwelling Nationalities, Guangxi, China; 4 The Second Clinical Medical College, Zhejiang Chinese Medical University, Hangzhou, Zhejiang, China

**Keywords:** climate change, ecological study, female infertility, heat stress, male infertility

## Abstract

**Background:**

Repeated exposure to heat stress may adversely affect reproductive health, yet the population-level relationship between heat stress and infertility has not been well characterized.

**Methods:**

We conducted a global ecological analysis across 174 countries and regions from 1990 to 2021. Heat exposure was quantified as the annual number of days falling into four Universal Thermal Climate Index categories: moderate, strong, very strong, and extreme heat stress. We additionally constructed a weighted score combining very strong and extreme heat stress. Associations with male and female infertility prevalence were estimated using linear mixed-effects models.

**Results:**

Each additional day of very strong and extreme heat stress was associated with increases in male infertility ASPR of 1.292 (95% confidence interval [CI]: 0.675–1.908) and 1.654 (95% CI: 0.214–3.096) per 100,000 population, respectively. The corresponding increases in female infertility ASPR were 2.126 (95% CI: 0.785–3.467) and 7.133 (95% CI: 3.940–10.323) per 100,000 population, respectively. Higher weighted heat-stress scores were also consistently associated with greater infertility prevalence.

**Conclusions:**

These findings suggest that recurrent heat-stress exposure may be associated with a higher population burden of infertility and support consideration of reproductive health in climate-change adaptation and heat-protection strategies.

## 1 Introduction

Infertility has become a substantial global public health concern. It affects approximately 10%−15% of couples of reproductive age worldwide, and the global fertility rate continues to exhibit a persistent downward trend (1). Many countries and regions are approaching or have already fallen below the replacement-level fertility threshold, raising concerns about potential demographic imbalances and socioeconomic consequences (2). The decline in fertility reflects the complex interaction of multiple determinants of fertility (3).

Infertility is a multifactorial reproductive health condition that may involve abnormalities occurring at multiple stages of the reproductive process. Female infertility may be associated with abnormalities in ovulation, ovarian function, oocyte quality, and early embryonic development (4, 5). Meanwhile, testicular dysfunction and impaired spermatogenesis may affect sperm quantity and quality, thereby being associated with reduced male fertility (6, 7). Growing evidence suggests that environmental exposures, including climate change and air pollution, are associated with reproductive health disorders (8, 9). In particular, the increasing occurrence of extreme heat events under global warming has received growing attention as an emerging environmental challenge to human health. Global warming has been accompanied by increases in the frequency, intensity, and duration of heatwaves, resulting in elevated population-level heat exposure (10). Existing studies suggest that high-temperature exposure may be associated with reduced sperm quality in men, altered ovarian function in women, and changes in assisted reproductive outcomes. Furthermore, heat stress may influence key reproductive processes, including fertilization, embryonic development, and implantation, through pathways involving cellular stress responses and altered reproductive tissue function (11, 12). Collectively, these findings provide biological plausibility for investigating the association between heat stress and reproductive health outcomes.

However, large-scale epidemiological investigations of the association between heat stress and infertility prevalence are limited. First, most previous investigations have relied on ambient temperature or simplified thermal indices as proxy measures of heat exposure, without adequately accounting for the combined influences of key meteorological variables on the actual thermal load experienced by the human body. Second, many existing observational studies are constrained by short observation periods and limited geographical scope, hindering the ability to elucidate long-term, large-scale epidemiological associations. Heat exposure varies in intensity, yet few studies have simultaneously investigated multiple levels of heat stress in relation to reproductive system disorders.

This study adopts the Universal Thermal Climate Index (UTCI) as the primary measure of heat exposure. It is widely recognized as a comprehensive and physiologically meaningful thermal index in the field of biometeorology (13–15). Owing to its physiological grounding and standardized modeling framework, UTCI demonstrates strong comparability and robustness across different climates, seasons, and spatiotemporal scales—from microenvironments to global assessments (16). Consequently, it has been extensively applied in public meteorological services, health warning systems, and climate impact assessments (17, 18).

Building upon this rationale, we conducted a global ecological study to examine the association between heat stress of varying intensities and male and female infertility age-standardized prevalence rates (ASPR). This study also aimed to elucidate the potential impacts of heat stress, within the context of ongoing climate change, on reproductive health. We hypothesized that an increase in the number of heat-stressed days would be positively associated with the increasing prevalence of infertility in recent years.

## 2 Methods

### 2.1 Sources of data

ERA5-HEAT is the first global historical gridded dataset developed using a human biometeorological index. Developed by the European center for medium-range weather forecasts (ECMWF) and made available through the Copernicus Climate Data Store, it provides hourly UTCI estimates at a spatial resolution of 0.25° × 0.25° on a regular latitude–longitude grid. The dataset extends from 1979 to the present and, based on ERA5, provides continuously updated coverage across global land and ocean areas (19). For the present study, global UTCI raster data covering 1990–2021 were obtained. Given the availability of administrative boundary shapefile data for 174 GBD countries and regions, the ecological analysis in this study is limited to these areas (Table S1 and Figure S1).

The GBD study provides a standardized framework and comparable data for quantifying health losses attributable to hundreds of diseases, injuries, and their associated risk factors (20). For this study, data on the ASPR of male and female infertility were extracted from GBD 1990–2021 and integrated with national-level estimates of ambient air pollution exposure, including PM, NO_2_, and O_3_, as well as the socio-demographic index (SDI).

### 2.2 Assessment of UTCI

UTCI is a human biometeorological index that quantifies thermal stress by expressing the thermal conditions of a target environment as an equivalent air temperature under a standardized reference environment. The UTCI value represents the temperature at which the same physiological response would occur under reference conditions. More information on the UTCI calculation method is provided in Text S1. According to established biometeorological classification criteria, UTCI values can be categorized into four heat stress intensity levels: moderate (26–32 °C), strong (32–38 °C), very strong (38–46 °C), and extreme (>46 °C) heat stress (Table S2 and Figure S2) (15). For each ERA5-HEAT grid cell, the annual number of days on which the daily maximum UTCI fell within each heat stress category was calculated in our study. Annual UTCI heat stress days were then aggregated at the country/region level by averaging the corresponding grid-cell values within each national boundary.

### 2.3 Assessment of infertility prevalence

Within the GBD 2021 framework, infertility was conceptualized as a couple level reproductive outcome and estimated using available epidemiological data, including fertility related information from population based reproductive surveys, mainly among married or cohabiting women. Because these surveys do not directly identify the responsible partner, the GBD DisMod MR 2.1 Bayesian meta regression framework integrates available evidence to generate sex specific estimates of male and female infertility prevalence (21, 22). These estimates were informed by epidemiological evidence, including sex attribution patterns from previous GBD analyses. Male and female infertility were analyzed as separate GBD defined outcomes, with corresponding ICD codes provided in Table S3 (23). Age standardized prevalence rates (ASPRs) were used as the primary outcomes.

### 2.4 Assessment of covariates

Country level covariates included the Socio demographic Index (SDI) and ambient air pollution exposures, including PM, NO_2_, and O_3_. These covariates were selected based on previous evidence and the assumed causal structure illustrated in the directed acyclic graph (DAG) (Figure S3). SDI is a composite indicator calculated as the geometric mean of three equally weighted components: total fertility rate among women younger than 25 years, average educational attainment among individuals aged 15 years and older, and lag distributed income per capita. Methods used to estimate ambient air pollution exposure have been reported previously (24).

### 2.5 Statistical analysis

We combined the numbers of days with moderate, strong, very strong, and extreme heat stress to define overall heat stress (days with UTCI ≥ 26 °C). Geodetector was applied to investigate the spatial association between the number of heat-stress days and infertility ASPR for both males and females, with a higher q-statistic indicating stronger spatial explanatory power (25). A detailed description of the application of Geodetector is provided in Text S2. To meet the methodological requirements of Geodetector, all variables were discretized into four categories using the Jenks natural breaks method. As sensitivity analyses, the variables were additionally classified into five and six categories using the same Jenks natural breaks approach, and the analyses were repeated to assess the robustness of the results.

To rank the relative importance of UTCI ≥ 26 °C, ambient air pollution exposure, and SDI, we fitted an XGBoost machine-learning model and interpreted the model using SHapley Additive exPlanations (SHAP). The XGBoost model was trained on country-year data (*N* = 5,568) using leave-one-country-out cross-validation to prevent information leakage across observations from the same country (26, 27). Based on Shapley values, SHAP assigns an additive contribution to each predictor, which allows variable importance to be ranked (28, 29).

We used linear mixed-effects models to evaluate the associations between the annual heat-stress days at different levels and infertility prevalence. Country/region was specified as a random intercept to account for repeated observations within each location. The models were adjusted for SDI, PM, NO_2_, and O_3_, with calendar year additionally included as a fixed effect to capture temporal trends common across countries/regions. Potential multicollinearity among the covariates was assessed using variance inflation factors (VIFs). The findings indicated that multicollinearity might not be a major concern in the present study, with VIFs ranging from 1.56 to 2.91 (Table S4). Associations were quantified using regression coefficients (βs) and corresponding 95% confidence intervals (CIs) derived from the multivariable-adjusted models.

To characterize the combined burden of very strong and extreme heat stress, we derived a weighted heat-stress score based on the estimated effects of these two exposure categories. The score was defined as β(very strong heat stress) × days of very strong heat stress + β(extreme heat stress) × days of extreme heat stress, with larger values representing greater cumulative heat-stress exposure. Previous studies have employed this method (30, 31). To limit potential overfitting arising from estimation of the weights in the same dataset, we implemented a cross-fitting strategy (32, 33). Countries/regions were randomly partitioned into 10 subsets. For each iteration, nine subsets were used as the training sample to estimate the regression coefficients used as weights, whereas the remaining subset served as the validation sample in which the weighted score was calculated. This procedure was repeated across all 10 subsets so that each subset was used once for validation, after which the out-of-sample scores were pooled to obtain the final score for the full dataset. Linear mixed-effects models were subsequently fitted to estimate the change in infertility prevalence associated with a one-unit increase in the weighted score. For subgroup analysis, stratification was performed based on SDI, continents and latitude.

A series of sensitivity analyses were performed to examine the stability of the associations between different levels of heat stress and infertility prevalence. Specifically, we: (1) refitted the models using a two -way fixed-effects specification with country and year fixed effects and Driscoll–Kraay standard errors to accommodate serial correlation and cross-sectional dependence; (2) repeated the analyses after log-transforming infertility prevalence to improve the normality of the outcome distribution; (3) additionally adjusted for country-level population density and repeated the analyses under two contrasting population–land area scenarios. Countries were first classified into tertiles (Q1–Q3) according to population density and land area. Scenario 1 included countries with population density in the highest tertile (Q3) and land area in the lowest tertile (Q1; *N* = 34), whereas Scenario 2 included countries with population density in the lowest tertile (Q1) and land area in the highest tertile (Q3; *N* = 35); (4) further adjusted for life expectancy, urbanization rate, and smoking prevalence to account for potential residual confounding related to healthcare conditions, urbanization, and lifestyle factors; (5) conducted a 0–5 year lag analysis to evaluate potential delayed associations between heat-stress exposure and infertility prevalence; and (6) evaluated temporal heterogeneity by comparing 1990–2009 with 2010–2021 using interaction terms between each heat-stress level and a post-2010 indicator. Statistical analyses were conducted in R (version 4.5.1) and ArcGIS (version 10.8), and statistical significance was defined using a two-sided *P*-value < 0.05.

## 3 Results

### 3.1 Characteristics of 174 countries/regions from 1990 to 2021

From 1990 to 2021, the global average (SD) annual number of days with UTCI ≥ 26 °C was 239.84 days (126.95). The mean ASPR for male and female infertility were 1115.89 per 100,000 population (518.03) and 1772.12 per 100,000 population (1038.51), respectively (Table 1). Regarding other environmental exposures is provided in the Table 2.

**Table 1:** Characteristics of UTCI ≥ 26 °C and male and female infertility ASPR by SDI, continent, and latitude in 1990–2021.

Variables	UTCI≥26 °C (days)	Infertility prevalence (per 100,000 population)	
		Male	Female
Global	239.84 (126.95)	1115.89 (518.03)	1772.12 (1038.51)
SDI			
Low	326.99 (64.28)	1256.95 (680.33)	1869.63 (1144.35)
Low-middle	274.12 (101.39)	1028.80 (447.80)	1823.96 (1065.25)
Middle	216.73 (113.56)	1101.34 (345.42)	1931.71 (878.50)
High-middle	140.33 (111.77)	1096.31 (388.34)	1670.77 (933.20)
High	64.80 (79.18)	919.79 (328.53)	1210.79 (757.56)
Continent			
Asia	234.97 (111.49)	960.85 (313.83)	1708.57 (924.70)
Europe	64.09 (43.09)	1064.40 (391.53)	1657.27 (814.52)
Africa	323.69 (56.84)	1336.09 (695.74)	2002.86 (1182.70)
North America	313.71 (89.43)	1133.83 (435.24)	1883.10 (1055.55)
South America	270.75 (95.21)	922.00 (442.18)	1246.69 (1066.24)
Oceania	292.98 (105.88)	993.79 (279.45)	1634.84 (1024.17)
Latitude			
Low	327.85 (53.39)	1179.70 (593.09)	1922.87 (1118.81)
Middle	104.22 (63.75)	999.78 (305.50)	1513.88 (807.44)
High	12.01 (9.12)	1128.25 (558.29)	1640.91 (1069.08)

UTCI ≥ 26 °C (Total number of days with moderate, strong, very strong, and extreme heat stress).

**Table 2:** Characteristics of environmental factors by SDI, continent, and latitude in 1990–2021.

Variables	PM (ug/m^3^)	Ozone (ppb)	NO_2_ (ppb)
Global	28.77 (16.88)	40.08 (9.79)	5.50 (3.88)
SDI			
Low	37.83 (18.02)	37.50 (9.26)	2.63 (1.56)
Low-middle	28.41 (13.77)	39.74 (10.81)	4.83 (2.81)
Middle	25.45 (12.78)	41.12 (9.71)	6.53 (3.54)
High-middle	22.62 (15.32)	43.26 (9.10)	8.84 (4.03)
High	14.97 (11.63)	42.37 (7.18)	9.41 (3.52)
Continent			
Asia	35.40 (17.70)	47.90 (9.84)	7.04 (4.04)
Europe	18.41 (7.42)	42.91 (5.32)	8.53 (2.97)
Africa	36.34 (17.94)	37.14 (7.83)	2.67 (1.71)
North America	21.13 (6.11)	37.30 (6.76)	5.04 (4.00)
South America	27.02 (16.96)	31.16 (4.66)	6.28 (2.28)
Oceania	11.84 (3.22)	23.82 (6.04)	1.69 (2.26)
Latitude			
Low	33.13 (18.40)	37.06 (10.05)	3.70 (3.07)
Middle	22.81 (10.46)	45.67 (6.89)	8.41 (3.18)
High	9.87 (4.42)	38.53 (2.74)	8.69 (3.57)

At the global level, the distribution of heat-stress days showed a clear latitudinal pattern. Heat-stress days were more frequent in tropical and subtropical areas, while temperate and higher-latitude countries generally experienced fewer days each year. The geographic distributions of both female and male infertility ASPR indicated relatively high prevalence in the Caribbean, sub-Saharan Africa, Europe, and East Asia (Figures 1A, B). Although the spatial distribution of infertility ASPR did not necessarily correspond directly to that of heat-stress days, relatively high ASPR was frequently observed in regions with greater heat-stress exposure.

**Figure 1:**
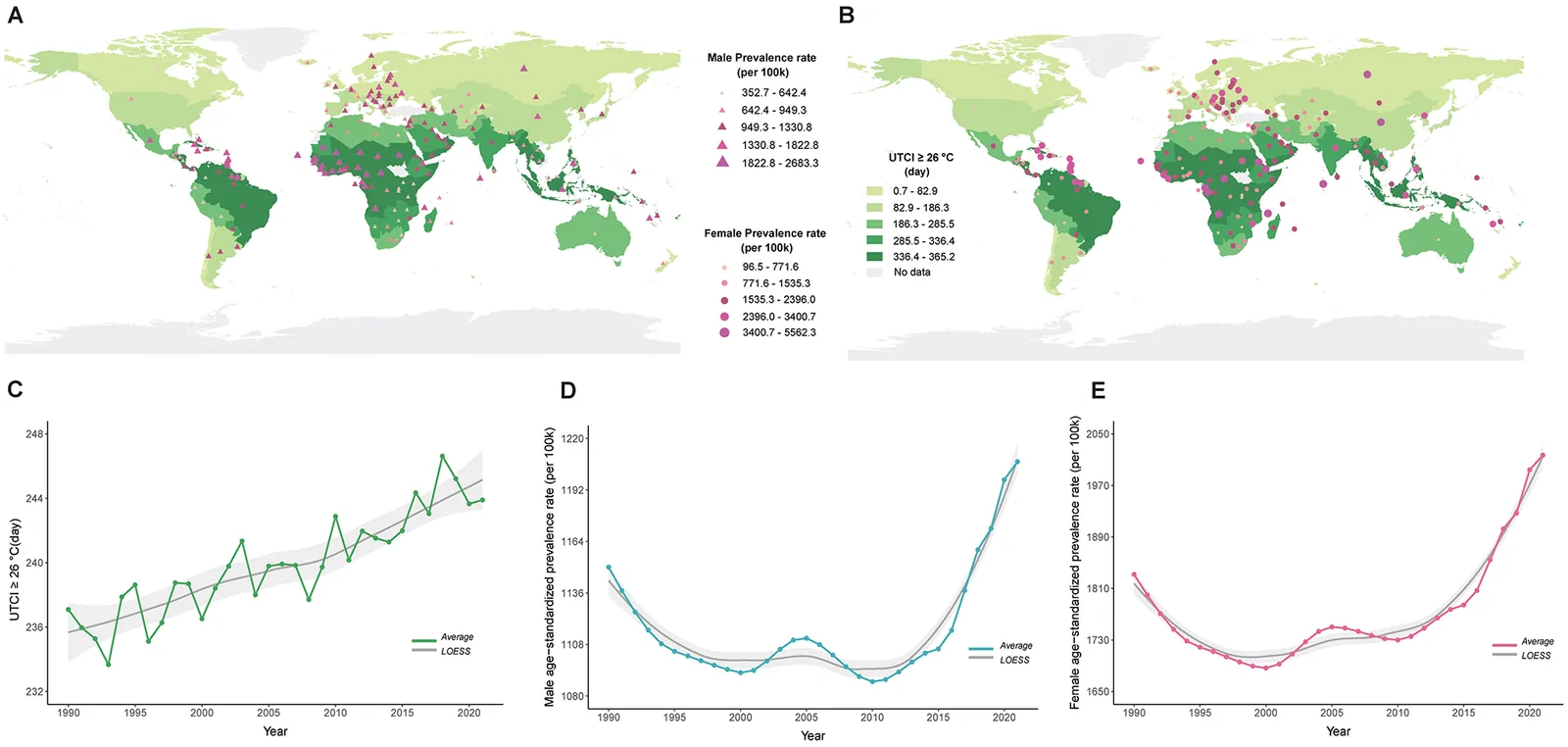
Descriptive distributions and trends of UTCI ≥ 26 °C and male and female infertility prevalences from 1990–2021. **(A, B)** Global distributions of the average annual number of days with UTCI ≥ 26 °C during 1990–2021, shown as background shading across five intervals. Overlaid symbols indicate the mean age-standardized prevalence rates (ASPRs) of male infertility **(A)** and female infertility **(B)** across countries. **(C)** Change in the number of days with UTCI ≥ 26 °C from 1990–2021. **(D, E)** Change in global ASPRs for male **(D)** and female **(E)** infertility from 1990–2021. LOESS, locally estimated scatterplot smoothing; UTCI ≥ 26 °C represents the total number of moderate, strong, very strong, and extreme heat stress days; UTCI, Universal Thermal Climate Index.

The annual average number of days with UTCI ≥ 26 °C generally increased over the study period (Figure 1C). The prevalence of male and female infertility fluctuated between 1990 and 2010; however, since 2010, both have exhibited a rapid upward trend (Figures 1D, E).

### 3.2 UTCI ≥ 26 °C and infertility prevalence

Geodetector analysis indicated spatial associations between the number of heat-stress days and the ASPR of both male and female infertility, with q-statistics of 0.100 and 0.094, respectively (both *P* < 0.05, Figures 2A, B). The results remained stable when the variables were alternatively classified into five and six categories using the Jenks natural breaks method (Table S5).

**Figure 2:**
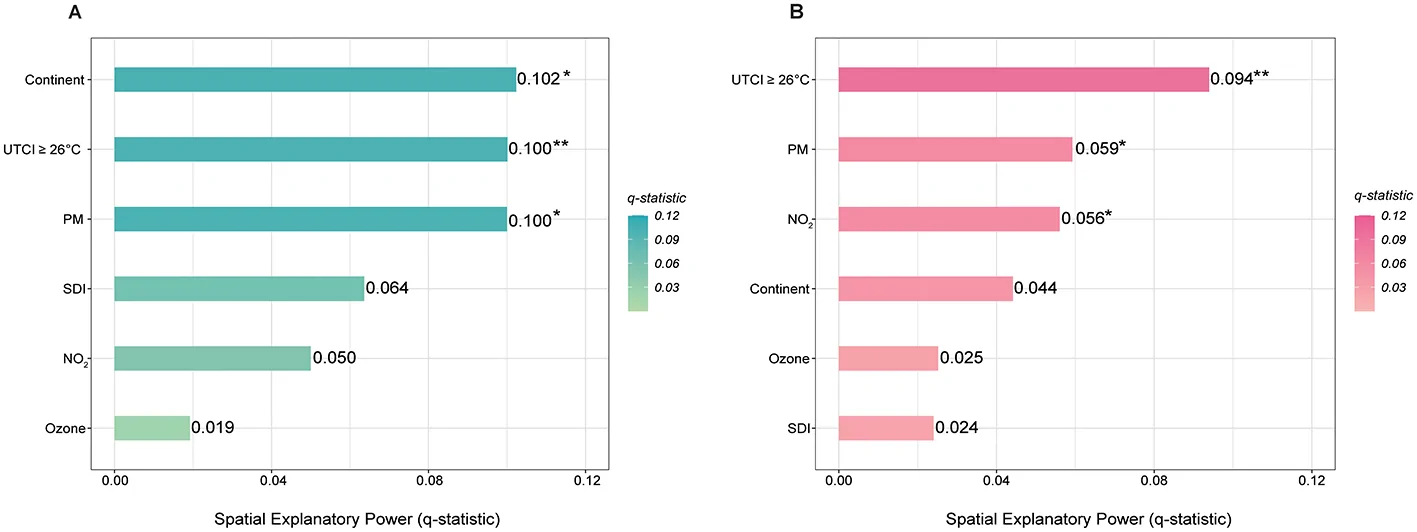
Spatial association between the average number of days with UTCI ≥ 26 °C and mean male **(A)** and female **(B)** infertility prevalences, 1990–2021. **P < 0.001, *P < 0.05. ASPRs, age-standardized prevalence rates; UTCI, Universal Thermal Climate Index.

In addition, SHAP analysis was performed to rank the relative importance of UTCI ≥ 26 °C, ambient air pollutant exposures, and SDI. Overall, the number of heat-stress days showed relatively high importance in explaining variation in the ASPR of both female and male infertility (Figure 3).

**Figure 3:**
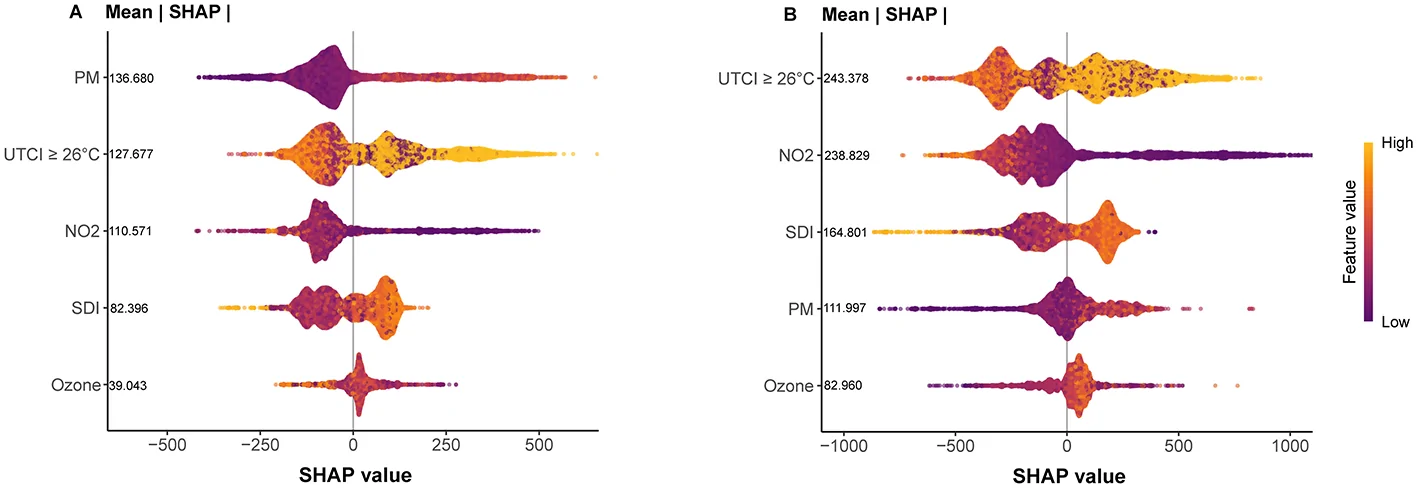
SHAP summary plot of male **(A)** and female **(B)** infertility. Variables are ranked by importance, based on the mean absolute SHAP value, from top to bottom. Each dot represents one observation; the x-axis shows the SHAP value. Positive values increase the model predicted infertility prevalence, whereas negative values decrease it. Dot color indicates the feature value for each observation. SHAP, SHapley Additive exPlanations.

### 3.3 Heat stress level and infertility prevalence

For the specific heat-stress levels, from 1990 to 2021, the mean (SD) annual numbers of days with moderate, strong, very strong, and extreme heat stress were 80.72 (62.99), 108.85 (88.91), 43.45 (55.68), and 6.82 (20.76), respectively (Table S6).

Greater numbers of days with very strong and extreme heat stress were associated with higher ASPR of both male and female infertility. Specifically, each additional day of very strong and extreme heat stress was associated with increases in male infertility ASPR of 1.292 (95% CI: 0.675–1.908) and 1.654 (95% CI: 0.214–3.096) per 100,000 population, respectively. The corresponding increases in female infertility ASPR were 2.126 (95% CI: 0.785–3.467) and 7.133 (95% CI: 3.940–10.323) per 100,000 population, respectively (Table 3). Moderate heat stress was positively associated with female infertility ASPR, whereas no statistically significant association was observed for male infertility. No statistically significant association with strong heat stress was observed for either male or female infertility.

**Table 3:** Association of heat stress levels with the prevalence of male and female infertility (per 100,000 population).

Heat stress level	Male		Female		*P* for interaction
	β (95% CI)	*P*	β (95% CI)	*P*	
Moderate heat stress	0.531 (−0.002, 1.063)	0.051	1.185 (0.014, 2.356)	0.048	< 0.001
Strong heat stress	0.362 (−0.096, 0.819)	0.122	−0.109 (−1.102, 0.882)	0.829	< 0.001
Very strong heat stress	1.292 (0.675, 1.908)	< 0.001	2.126 (0.785, 3.467)	0.002	< 0.001
Extreme heat stress	1.654 (0.214, 3.096)	0.025	7.133 (3.940, 10.323)	< 0.001	< 0.001

Adjustments were made for SDI, PM, NO_2_, and O_3._

### 3.4 Weighted heat stress score and infertility prevalence

A higher heat-stress score, reflecting the weighted contributions of very strong and extreme heat stress, was positively associated with infertility prevalence. For each one-unit increase in the heat-stress score, male and female infertility prevalence increased by 0.337 (95% CI: 0.012–0.662) and 0.909 (95% CI: 0.590–1.228) per 100,000 population, respectively (Figure 4).

**Figure 4:**
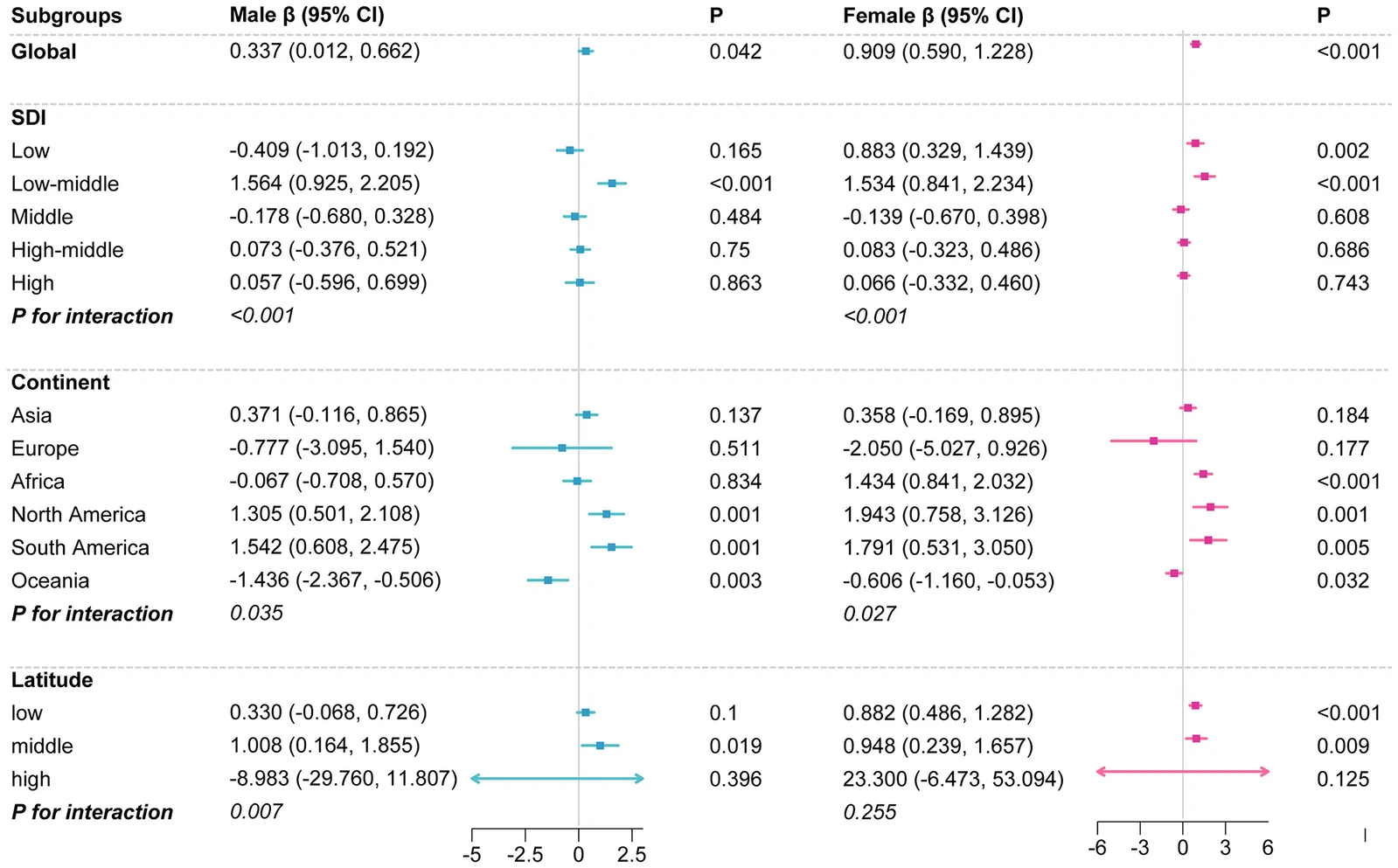
Subgroup analysis results of the associations between the heat stress score and prevalence of male and female infertility. Adjustments were made for the SDI, PM, NO_2_ and O_3_. Heat stress score (weighted scores of very strong and extreme heat stress). Models for subgroup analyses across categorical variables were not adjusted for the stratification factor itself. SDI, socio-demographic index.

Subgroup analyses indicated effect modification by SDI and continent (all *P* for interaction < 0.05), whereas the interaction with latitude remained significant only among males (Figure 4). These interaction findings remained unchanged after false discovery rate (FDR) correction (Table S7). In analyses stratified by SDI, significant associations between the heat-stress score and female infertility prevalence were observed in the low- and low-middle-SDI groups. In continent-stratified analyses, significant associations were observed in Africa, North America, and South America. Similarly, in analyses stratified by latitude, significant associations were observed in the low- and middle-latitude groups. Broadly similar patterns were observed for male infertility prevalence (Figure 4).

### 3.5 Sensitivity analysis

Overall, the results of the sensitivity analysis remain consistent. The associations between heat stress and infertility prevalence remained directionally stable after applying two-way fixed-effects models with Driscoll–Kraay standard errors, log-transforming infertility prevalence, additionally adjusting for population density and other covariates, restricting the analyses to contrasting population density–land area scenarios, and accounting for delayed effects (Tables S8, S9). Period-specific analyses further showed that most associations did not differ significantly between 1990–2009 and 2010–2021, although significant temporal heterogeneity was observed for some heat-stress levels (Table S10).

## 4 Discussion

In this study, we examined heat stress and a composite heat stress score with infertility prevalence. A higher number of very strong and extreme heat stress days was associated with increased prevalence of both male and female infertility, with the association appearing stronger for female infertility. Consistently, higher heat stress scores were also related to greater infertility prevalence in both sexes. These associations tended to be more pronounced in low and low-middle SDI regions and in areas located at low to middle latitudes.

Our findings align broadly with existing evidence regarding ambient temperature and human reproductive health. Nevertheless, epidemiological evidence on the relationship between heat stress and infertility at a large population scale remains scarce. A nationwide pre-conception cohort study involving over 14 million Chinese couples planning a pregnancy found that heat stress exposure during the pre-conception period was associated with reduced fertility (34). Specifically, heat stress exposure was linked to a longer time to pregnancy, suggesting that physiologically significant heat stress may adversely affect human reproductive capacity. However, that study focused primarily on the pre-conception period and was limited to a single country. Building upon this, we assessed the long-term association of annual heat-stress exposure with infertility prevalence across a wider range of countries and regions. Furthermore, a scoping review by Heo et al., which included 135 epidemiological studies, revealed that previous research utilized diverse temperature exposure metrics, such as ambient temperature, heat indices, and extreme temperature events (35). The review noted significant heterogeneity and a reliance on single metrics for exposure assessment, alongside a lack of granular temperature exposure evaluation. Consequently, there remains a lack of clarity regarding whether varying levels of heat stress correspond to different degrees of impact on reproductive health. In the present study, we observed that very strong and extreme heat stress were associated with increased infertility prevalence in both male and female, whereas associations with lower levels of heat stress were less pronounced; this suggests that the link between heat stress and infertility prevalence may be more evident under conditions of higher heat stress level. By simultaneously evaluating different heat stress levels based on the physiologically defined UTCI, our study provides a more refined characterization of heat stress compared to studies relying solely on ambient temperature or single extreme heat metrics, thereby facilitating the identification of associations between infertility and specific levels of heat stress. Additionally, we constructed a composite heat stress score to reflect the overall exposure burden of high-intensity heat stress; higher scores were positively associated with increased infertility prevalence in both male and female, further indicating that high-intensity heat stress may be a scenario warranting greater attention in the context of reproductive health.

These findings are broadly consistent with previous research on high temperature exposure and reproductive health in both male and female. Several studies have reported that exposure to high ambient temperatures and heatwaves is associated with poorer sperm quality, including lower sperm counts, reduced motility, and a higher prevalence of abnormal morphology. These findings suggest that elevated temperatures may impair male reproductive capacity by disrupting spermatogenesis (36). Similar concerns have also been raised for female reproductive health, as higher ambient temperatures have been associated with reduced antral follicle counts and diminished ovarian reserve, potentially affecting follicular development and ovarian function (5, 37). Furthermore, existing studies suggest the potential existence of specific heat-sensitive pathways within the female reproductive system. Heat stress has been linked not only to reduced fertility but also to abnormalities in thyroid stimulating hormone levels in female, indicating that endocrine alterations may contribute to the reproductive effects of heat exposure (34). Beyond these biological mechanisms, women in certain contexts may also face greater heat and dehydration burdens due to gender-specific patterns of daily activity (38, 39), for example through unpaid household labor and water-fetching responsibilities (40, 41). These findings may help explain the stronger associations observed among women.

This study also identified regional variations in the magnitude of associations between heat stress and infertility. Differences in adaptive capacity may contribute to these regional variations. Higher SDI regions generally have better access to cooling infrastructure, healthcare services, and health related resources, which may reduce the adverse health effects associated with heat exposure. In contrast, populations in lower SDI areas may experience greater challenges in accessing protective measures, potentially increasing susceptibility to heat exposure (42). Additionally, lower SDI regions often experience a greater burden of malnutrition and other health-related challenges, which may coexist with heat exposure and contribute to differences in reproductive health outcomes (43). Interestingly, the strongest association was observed in low-middle SDI regions rather than low SDI regions. This may be because infertility related to factors such as infections may account for a larger proportion of the overall infertility burden in low SDI regions, potentially reducing the relative contribution of environmental heat exposure (44, 45). The negative correlation observed in Oceania may stem from a combination of factors. First, the relatively small number of Oceanian countries included in the analysis may reduce the stability of the estimates and render the results more susceptible to the influence of individual nations. There is significant heterogeneity across Oceania and the Pacific Island nations regarding geographical settings, population distribution, socioeconomic conditions, and health system capacity; furthermore, remote and dispersed population patterns may limit access to reproductive health and fertility services, potentially influencing regional-level effect estimates (46, 47). Additionally, for geographically dispersed island nations, national-level average heat exposure may not adequately reflect actual population exposure levels. Residual confounding, as well as uncertainties in the estimation of ecological exposure and health outcomes, may also contribute to this result.

From a public health perspective, our findings contribute to the emerging evidence on the potential reproductive health implications of increasing heat exposure under ongoing global warming. The frequency and intensity of very strong and extreme heat stress events are projected to increase in the future, which may further highlight the importance of understanding the potential implications of heat exposure for reproductive health, particularly in vulnerable regions (48). More broadly, our findings emphasize the need to consider reproductive health outcomes in climate adaptation strategies and public health planning.

## 5 Strengths and limitations of this study

The primary strength of this ecological study lies in its global scale, extended temporal coverage, and the use of the physiologically based UTCI index to finely classify heat exposure. Nevertheless, several limitations should be considered when interpreting the findings. First, given the ecological nature of this study, the associations observed between heat-stress exposure and infertility should be interpreted at the population level rather than at the individual level, and no causal relationship can be established. Second, although adjustments were made for national SDI and major air pollutants, residual confounding cannot be completely excluded. Although additional sensitivity analyses incorporating smoking prevalence, urbanization rate, and life expectancy showed consistent results, unavailable or inconsistently measured factors, including assisted reproductive technology accessibility and occupational heat exposure, may still influence the observed associations. Third, infertility estimates are derived from population-based reproductive surveys and modeled epidemiological evidence rather than individual clinical assessments. Although GBD provides standardized estimates for global comparisons, differences in definitions and diagnostic criteria across countries and regions may introduce uncertainty and affect comparability. Future studies incorporating individual-level cohort designs, higher resolution exposure assessments, and more comprehensive covariate information are warranted to further validate these findings and clarify the underlying mechanisms. Fourth, heat stress exposure was summarized at the national level, which may not adequately reflect spatial variation within individual countries. This spatial averaging could introduce exposure misclassification and potentially bias the estimated. Fifth, the study was based on globally standardized, model-derived datasets. While these data allowed comparable estimates to be obtained across countries and over an extended period, they may still be subject to uncertainty from the underlying models and differences in data quality between countries. Accordingly, the results should be viewed as ecological evidence at the country/region level, and caution is warranted when extrapolating the findings across different national contexts.

## 6 Conclusion

This study demonstrates that increased exposure to very strong and extreme heat stress, as defined by the UTCI, is significantly associated with higher age-standardized prevalence rates of male and female infertility. The associations were stronger for female infertility and among populations in lower SDI regions. The application of comprehensive thermal indices such as UTCI provides more refined evidence for understanding heat related reproductive health challenges and may support the development of climate adaptation strategies and public health planning aimed at reducing heat stress exposure and addressing its potential implications for reproductive health.

## Data Availability

The original contributions presented in the study are included in the article/Supplementary material, further inquiries can be directed to the corresponding authors.

## References

[1] Gnoth C Godehardt E Frank-Herrmann P Friol K Tigges J Freundl G. Definition and prevalence of subfertility and infertility. Hum Reprod. (2005) 20:1144–7. doi: 10.1093/humrep/deh870 15802321

[2] Wilson C. Fertility below replacement level. Science. (2004) 304:207–9. doi: 10.1126/science.304.5668.207c 15073356

[3] Carson SA Kallen AN. Diagnosis and management of infertility: a review. JAMA. (2021) 326:65–76. doi: 10.1001/jama.2021.4788 PMC9302705 34228062

[4] Sun XF Wang WH Keefe DL. Overheating is detrimental to meiotic spindles within *in vitro* matured human oocytes. Zygote. (2004) 12:65–70. doi: 10.1017/S0967199404002631 15214582

[5] Geng L Yang Y Chen Y Ye T Qiu A Bukulmez O . Association between ambient temperature exposure and pregnancy outcomes in patients undergoing *in vitro* fertilization in Shanghai, China: a retrospective cohort study. Hum Reprod. (2023) 38:2489–98. doi: 10.1093/humrep/dead192 37759343

[6] Liu B Wang J Liu L Lv M Zhou D Li M. Hyperbaric oxygen therapy for male infertility: a systematic review and meta-analysis on improving sperm quality and fertility outcomes. Med Gas Res. (2025) 15:529–34. doi: 10.4103/mgr.MEDGASRES-D-24-00153 PMC12124701 40300888

[7] Gao Y Wang C Wang K He C Hu K Liang M. The effects and molecular mechanism of heat stress on spermatogenesis and the mitigation measures. Syst Biol Reprod Med. (2022) 68:331–47. doi: 10.1080/19396368.2022.2074325 35722894

[8] Fernandez ACG Pelnekar S Robinson JF Shaw GM Padula AM Woodruff TJ . Climate change and reproductive health. Endocr Rev. (2025) 46:908–21. doi: 10.1210/endrev/bnaf026 PMC12640714 40755394

[9] Segal TR Giudice LC. Systematic review of climate change effects on reproductive health. Fertil Steril. (2022) 118:215–23. doi: 10.1016/j.fertnstert.2022.06.005 35878942

[10] Romanello M Walawender M Hsu SC Moskeland A Palmeiro-Silva Y Scamman D . The 2024 report of the Lancet Countdown on health and climate change: facing record-breaking threats from delayed action. Lancet. (2024) 404:1847–96. doi: 10.1016/S0140-6736(24)01492-2 PMC7616816 39488222

[11] Mahan VL. Heme oxygenase/carbon monoxide system and development of the heart. Med Gas Res. (2025) 15:10–22. doi: 10.4103/mgr.MEDGASRES-D-24-00031 PMC11515065 39324891

[12] Wang H Wang Z Wu Q Yang Y Liu S Bian J . Perioperative oxygen administration for adults undergoing major noncardiac surgery: a narrative review. Med Gas Res. (2025) 15:73–84. doi: 10.4103/mgr.MEDGASRES-D-24-00010 PMC11515063 39436170

[13] Błażejczyk K Broede P Fiala D Havenith G Holmér I Jendritzky G . Principles of the new Universal Thermal Climate Index (UTCI) and its application to bioclimatic research in European scale. Misc Geogr Reg Stud Dev. (2010) 14:91–102. doi: 10.2478/mgrsd-2010-0009

[14] Staiger H Laschewski G Matzarakis A. Selection of appropriate thermal indices for applications in human biometeorological studies. Atmosphere. (2019) 10:18. doi: 10.3390/atmos10010018

[15] Błażejczyk K Jendritzky G Bröde P Fiala D Havenith G Epstein Y . An introduction to the Universal Thermal Climate Index (UTCI). Geogr Pol. (2013) 86:5–10. doi: 10.7163/GPol.2013.1

[16] Jendritzky G De Dear R Havenith G. UTCI—why another thermal index? Int J Biometeorol. (2012) 56:421–8. doi: 10.1007/s00484-011-0513-7 22187087

[17] Zare S Hasheminejad N Shirvan HE Hemmatjo R Sarebanzadeh K Ahmadi S. Comparing Universal Thermal Climate Index (UTCI) with selected thermal indices/environmental parameters during 12 months of the year. Weather Clim Extremes. (2018) 19:49–57. doi: 10.1016/j.wace.2018.01.004

[18] Blazejczyk K Epstein Y Jendritzky G Staiger H Tinz B. Comparison of UTCI to selected thermal indices. Int J Biometeorol. (2012) 56:515–35. doi: 10.1007/s00484-011-0453-2 PMC3337419 21614619

[19] Di Napoli C Barnard C Prudhomme C Cloke HL Pappenberger F. ERA5-HEAT: a global gridded historical dataset of human thermal comfort indices from climate reanalysis. Geosci Data J. (2021) 8:2–10. doi: 10.1002/gdj3.102

[20] GBD 2021 Diseases and Injuries Collaborators. Global incidence, prevalence, years lived with disability (YLDs), disability-adjusted life-years (DALYs), and healthy life expectancy (HALE) for 371 diseases and injuries in 204 countries and territories and 811 subnational locations, 1990–2021: a systematic analysis for the Global Burden of Disease Study 2021. Lancet. (2024) 403:2133–61. 10.1016/S0140-6736(24)00757-8 PMC11122111 38642570

[21] Sun H Gong TT Jiang YT Zhang S Zhao YH Wu QJ. Global, regional, and national prevalence and disability-adjusted life-years for infertility in 195 countries and territories, 1990–2017: results from a Global Burden of Disease Study, 2017. Aging. (2019) 11:10952–91. doi: 10.18632/aging.102497 PMC6932903 31790362

[22] Qian J Si Y Chen Y Zhu Y Zhu J Mo C . Ambient temperature and female infertility prevalence: an ecological study based on the 2019 global burden of disease study. Reprod Biol Endocrinol. (2025) 23:27. doi: 10.1186/s12958-025-01365-4 PMC11846354 39987432

[23] Global Burden of Disease Collaborative Network. Global Burden of Disease Study 2021 (GBD 2021) causes of death and nonfatal causes mapped to ICD codes. Seattle, WA: Institute for Health Metrics and Evaluation (IHME) (2024).

[24] Global Burden of Disease Collaborative Network. Global Burden of Disease Study 2021 (GBD 2021) Air Pollution Exposure Estimates and Risk Curves 1990–2021. Seattle, WA: Institute for Health Metrics and Evaluation (IHME) (2024).

[25] Wang JF Xu CD. Geodetector: principle and prospective. Acta Geogr Sin. (2017) 72:116–34.

[26] Le Rest K Pinaud D Monestiez P Chadoeuf J Bretagnolle V. Spatial leave-one-out cross-validation for variable selection in the presence of spatial autocorrelation. Glob Ecol Biogeogr. (2014) 23:811–20. doi: 10.1111/geb.12161

[27] Airola A Pohjankukka J Torppa J Middleton M Nykänen V Heikkonen J . The spatial leave-pair-out cross-validation method for reliable AUC estimation of spatial classifiers. Data Min Knowl Discov. (2019) 33:730–47. doi: 10.1007/s10618-018-00607-x

[28] Lundberg SM Lee SI A. unified approach to interpreting model predictions. Adv Neural Inf Process Syst. (2017) 30:4765–74.

[29] Lundberg SM Erion G Chen H DeGrave A Prutkin JM Nair B . From local explanations to global understanding with explainable AI for trees. Nat Mach Intell. (2020) 2:56–67. doi: 10.1038/s42256-019-0138-9 PMC7326367 32607472

[30] Li FR Zhu B Liao J Cheng Z Jin C Mo C . Ambient air pollutants and incident microvascular disease: a cohort study. Environ Sci Technol. (2022) 56:8485–95. doi: 10.1021/acs.est.2c00898 35616623

[31] Wang M Zhou T Song Y Li X Ma H Hu Y . Joint exposure to various ambient air pollutants and incident heart failure: a prospective analysis in UK Biobank. Eur Heart J. (2021) 42:1582–91. doi: 10.1093/eurheartj/ehaa1031 PMC8060055 33527989

[32] Chernozhukov V Chetverikov D Demirer M Duflo E Hansen C Newey W . Double/debiased machine learning for treatment and structural parameters. Econom J. (2018) 21–C68. doi: 10.1111/ectj.12097

[33] Zivich PN Breskin A. Machine learning for causal inference: on the use of cross-fit estimators. Epidemiology. (2021) 32:393–401. doi: 10.1097/EDE.0000000000001332 PMC8012235 33591058

[34] He X Li M Yang Y Lv X Xu H Zhang B . Environmental heat and cold stress, thyrotropin abnormalities, and reduced fecundability in 14 million Chinese couples: a nationwide preconception cohort study. Environ Res. (2026) 298:124192. doi: 10.1016/j.envres.2026.124192 41791472

[35] Heo S Byun G Choi Y Song Y Bravo M Ma T . A scoping review on the impact of ambient temperature on human infertility. Environ Res. (2026) 292:123641. doi: 10.1016/j.envres.2025.123641 PMC12820886 41456660

[36] Zhou Y Meng T Wu L Duan Y Li G Shi C . Association between ambient temperature and semen quality: a longitudinal study of 10 802 men in China. Environ Int. (2020) 135:105364. doi: 10.1016/j.envint.2019.105364 31841801

[37] Gaskins AJ Mínguez-Alarcón L VoPham T Hart JE Chavarro JE Schwartz J . Impact of ambient temperature on ovarian reserve. Fertil Steril. (2021) 116:1052–60. doi: 10.1016/j.fertnstert.2021.05.091 PMC8478715 34116830

[38] Wickham KA McCarthy DG Spriet LL Cheung SS. Sex differences in the physiological responses to exercise-induced dehydration: consequences and mechanisms. J Appl Physiol. (2021) 131:504–10. doi: 10.1152/japplphysiol.00266.2021 34197234

[39] Yanovich R Ketko I Charkoudian N. Sex differences in human thermoregulation: relevance for 2020 and beyond. Physiology. (2020) 35:177–84. doi: 10.1152/physiol.00035.2019 32293229

[40] Sidun NM Gibbons JL. Women, girls, and climate change: human rights, vulnerabilities, and opportunities. Int J Psychol. (2024) 59:257–66. doi: 10.1002/ijop.12942 37696777

[41] Butler-Dawson J Jaramillo D Krisher L Hernandez KV Calvimontes L Dally M . A work and off-work evaluation of female workers' heat and particulate matter exposures and kidney health in Guatemala. J Clim Chang Health. (2025) 22:100408. doi: 10.1016/j.joclim.2024.100408 PMC12448058 40980813

[42] Pavanello F De Cian E Davide M Mistry M Cruz T Bezerra P . Air-conditioning and the adaptation cooling deficit in emerging economies. Nat Commun. (2021) 12:6460. doi: 10.1038/s41467-021-26592-2 PMC8578436 34753905

[43] GBD 2021 Risk Factors Collaborators. Global burden and strength of evidence for 88 risk factors in 204 countries and 811 subnational locations, 1990–2021: a systematic analysis for the Global Burden of Disease Study 2021. Lancet. (2024) 403:2162–203. 10.1016/S0140-6736(24)00933-4 PMC11120204 38762324

[44] Ombelet W Cooke I Dyer S Serour G Devroey P. Infertility and the provision of infertility medical services in developing countries. Hum Reprod Update. (2008) 14:605–21. doi: 10.1093/humupd/dmn042 PMC2569858 18820005

[45] Wei J Huang H Fan L. Global burden of female infertility attributable to sexually transmitted infections and maternal sepsis: 1990–2021 and projections to 2050. Sci Rep. (2025) 15:15189. doi: 10.1038/s41598-025-94259-9 PMC12043824 40307311

[46] Horwood PF Tarantola A Goarant C Matsui M Klement E Umezaki M . Health challenges of the Pacific region: insights from history, geography, social determinants, genetics, and the microbiome. Front Immunol. (2019) 10:2184. doi: 10.3389/fimmu.2019.02184 PMC6753857 31572391

[47] Patel C Sargent GM Tinessia A Mayfield H Chateau D Ali A . Measuring what matters: context-specific indicators for assessing immunisation performance in Pacific Island Countries and Areas. PLoS Glob Public Health. (2024) 4:e0003068. doi: 10.1371/journal.pgph.0003068 PMC11271932 39052626

[48] Horton RM Mankin JS Lesk C Coffel E Raymond C. A review of recent advances in research on extreme heat events. Curr Clim Change Rep. (2016) 2:242–59. doi: 10.1007/s40641-016-0042-x

