# Supplementary material for: Association between infertility prevalence and exposure to heat stress: an ecological study based on the Universal Thermal Climate Index
Source: Front Public Health. 2026 Oct 16;14:1948152. doi: 10.3389/fpubh.2026.1948152 (PMC13719622; doi:10.3389/fpubh.2026.1948152)
Supplement: Supplementary file 1 [file Data_Sheet_1.docx]

**Supplementary File Information**

**Text S1** More information for the UTCI calculation method.

**Text S2** Introduction to the use of geographic detectors

**Table S1** List of 174 countries/regions in each GBD region.

**Table S2** UTCI equivalent temperature categorized in terms of thermal stress.

**Table S3** List of International Classification of Diseases codes mapped to infertility in the GBD 2021.

**Table S4** Multicollinearity test for variables.

**Table S5** Spatial association between the average number of days with UTCI ≥ 26 °C and mean

male and female infertility prevalence under different Jenks classifications, 1990–2021.

**Table S6** Characteristics of UTCI ≥ 26°C and male and female infertility ASPR by SDI, continent, and latitude in 1990–2021

**Table S7** Subgroup interaction analyses for heat stress score and infertility prevalence with FDR-adjusted P values

**Table S8** Association between male and female infertility ASPR and heat stress levels (Sensitivity analysis 1)

**Table S9** Association between male and female infertility ASPR and heat stress levels (Sensitivity analyses 2–5)

**Table S10** Interaction tests for differences in the associations between heat stress levels and infertility prevalence before and after 2010 among male and female.

**Figure S1** Data screening process for the present study.

**Figure S2** The change in number of moderate heat stress, strong heat stress, very strong heat stress and extreme heat stress days from 1990 to 2021.

**Figure S3** Directed Acyclic Graph showing the assumed associations between heat stress, covariates, and infertility.

**Text S1 More information for the UTCI calculation method.**

UTCI was jointly developed by the International Society of Biometeorology and the European COST Action 730 program. It is based on the validated multi-node thermoregulation model (UTCI-Fiala) and advanced clothing models to simulate the equivalent temperature that would elicit the same physiological response in a reference environment.

UTCI quantifies heat exposure by expressing the temperature of a standard reference environment that would elicit the same human thermal response as the target environment as an equivalent air temperature. Compared with single-temperature metrics, UTCI more accurately reflects the body’s physiological responses to heat stress, such as variations in sweating and skin blood flow, thereby offering substantial advantages in evaluating the health impacts of environmental heat exposure. This value is physically equivalent to the air temperature in the reference environment. The reference activity is defined as walking at 4 km/h (metabolic rate ≈ 2.3 MET, or ~135 W·m⁻²), and the reference environment is specified as a wind speed of approximately 0.5 m·s⁻¹ at 10 m, mean radiant temperature equal to air temperature, and relative humidity of 50% (with water vapor pressure fixed at 20 hPa when Ta > 29 °C) [1]. By this approach, the multivariate thermal environment is normalized into a temperature-based index, expressed as: UTCI = Ta + Offset(Ta, Tmrt, va, RH).

The actual calculation employs an automated polynomial approximation of the UTCI-Fiala human thermoregulation model. When air temperature (Ta), mean radiant temperature (Tmrt), wind speed (va), and relative humidity (RH) are known, the equivalent temperature offset relative to Ta is obtained using a sixth-order polynomial that includes both main effects and interaction terms, yielding an average Root Mean Square Error of approximately 1.1 °C. This approximation is valid within the input ranges −50 °C ≤ Ta ≤ 50 °C, −30 °C ≤ (Tmrt − Ta) ≤ 70 °C, and RH ≤ 100%. To prevent non-physical extremes, the upper limit for wind speed is set at 17 m·s⁻¹, with values exceeding this range treated as missing [2].

Daily maximum UTCI values were extracted from ERA5-HEAT, and the number of days exceeding each of the four heat stress thresholds was calculated annually to serve as indicators of heat exposure at varying intensities. The resulting data were subsequently spatially aggregated to the national level using the administrative boundaries of GBD countries and regions.

Reference：

[1] K. Błażejczyk, G. Jendritzky, P. Bröde, D. Fiala, G. Havenith, Y. Epstein, A. Psikuta, B. Kampmann, An introduction to the universal thermal climate index (UTCI), Geogr. Pol. 86 (2013) 5–10. <https://doi.org/10.7163/GPol.2013.1>

[2] C. Di Napoli, C. Barnard, C. Prudhomme, H.L. Cloke, F. Pappenberger, ERA5‐HEAT: A global gridded historical dataset of human thermal comfort indices from climate reanalysis, Geosci. Data J. 8 (2021) 2–10. https://doi.org/10.1002/gdj3.102.

**Text S2 Introduction to the use of geographic detectors**

Spatial heterogeneity refers to the geographical phenomenon that the variance within a layer is smaller than the variance between layers. "Strata" is a statistical concept, roughly corresponding to geographical classes or sub regions. Heterogeneity and factor detection are used to detect the spatial heterogeneity of Y and to detect the degree of spatial correlation between factor X and attribute Y. Using the q value measurement, the expression is:

*h* = 1, …, *L* is the strata of variable *Y* or factor *X*; *N_h_* and *N* are the number of units in stratum *h* and the whole area respectively; and are the variances of stratum *h* and the *Y* value in whole area respectively. *SSW* and *SST* are the sum of variances within the stratum (Within Sum of Squares) and the total variance of the whole area (Total Sum of Squares), respectively. The range of *q* is [0, 1]. The larger the value, the more obvious the spatial heterogeneity of *Y* is; if the stratification is generated by the independent variable *X*, the larger the *q* value, the stronger the spatial correlation between the independent variable *X* and the attribute *Y*, and vice versa.

A simple transformation of the *q*-values satisfies the noncentral *F* distribution:

*λ* is the non-central parameter; is the mean of layer *h*. According to formula (3), we can test whether the q value is significant.

Reference：

[1] Jinfeng W. Chengdong X. Geodetector: principles and prospects. Acta Geogr Sin. 2017;72.

**Table S1** List of 174 countries/regions in each GBD region.

| East Asia | China, Korea (North) |
| --- | --- |
| Central Asia | Armenia, Azerbaijan, Georgia, Kazakhstan, Kyrgyzstan, Mongolia,  Tajikistan, Turkmenistan, Uzbekistan |
| South Asia | Bangladesh, Bhutan, India, Nepal, Pakistan |
| Southeast Asia | Cambodia, Indonesia, Laos, Malaysia, Maldives, Burma, Philippines,  Sri Lanka, Thailand, Timor-Leste, Mauritius, Seychelles |
| High-income Asia Pacific | Brunei, Japan, Korea (South) |
| Oceania | Fiji, Kiribati, Micronesia (Fed States of), Papua New Guinea,  Solomon Is, Vanuatu |
| Australasia | Australia, New Zealand |
| Andean Latin America | Bolivia, Ecuador, Peru |
| Tropical Latin America | Brazil, Paraguay |
| Central Latin America | Colombia, Costa Rica, El Salvador, Guatemala, Honduras, Mexico,  Nicaragua, Panama, Venezuela |
| Southern Latin America | Argentina, Chile, Uruguay |
| Caribbean | Antigua & Barbuda, Bahamas (The), Belize, Cuba, Dominican (Republic),  Grenada, Guyana, Haiti, Jamaica, St Lucia, St Vincent & the Grenadines,  Suriname, Trinidad & Tobago |
| High-income North America | Canada, United States |
| Central Europe | Albania, Bosnia & Herzegovina, Croatia, Czechia, Hungary, Macedonia,  Poland, Romania, Slovakia, Slovenia |
| Western Europe | Andorra, Austria, Belgium, Cyprus, Denmark, Finland, France, Germany,  Greece, Iceland, Ireland, Israel, Italy, Luxembourg, Netherlands, Norway,  Portugal, Spain, Sweden, Switzerland, United Kingdom |
| Eastern Europe | Belarus, Estonia, Latvia, Lithuania, Moldova, Russia, Ukraine |
| North Africa and Middle East | Algeria, Bahrain, Egypt, Iran, Iraq, Jordan, Kuwait, Lebanon, Libya,  Morocco, Oman, Qatar, Saudi Arabia, Syria, Tunisia, United Arab Emirates,  Yemen, Afghanistan, Sudan |
| Western Sub-Saharan Africa | Benin, Burkina Faso, Cameroon, Chad, Cote d'Ivoire, Gambia (The),  Ghana, Guinea, Liberia, Mali, Mauritania, Niger, Nigeria, Senegal,  Sao Tome & Principe, Sierra Leone, Togo |
| Eastern Sub-Saharan Africa | Burundi, Comoros, Djibouti, Eritrea, Ethiopia, Kenya, Madagascar,  Malawi, Mozambique, Rwanda, Somalia, Tanzania, Uganda, Zambia |
| Southern Sub-Saharan Africa | Botswana, Lesotho, Namibia, South Africa, Swaziland, Zimbabwe |
| Central Sub-Saharan Africa | Angola, Central African (Rep), Congo (Rep of the), Equatorial Guinea  Congo (Dem Rep of the), Gabon, Guinea-Bissau |

**Table S2** UTCI equivalent temperature categorized in terms of thermal stress.

| **UTCI (°C) range** | **Stress category** | **Physiological responses** |
| --- | --- | --- |
| above + 46 | extreme heat stress | – increase in rectal temperature time gradient  – steep decrease in total net heat loss  – averaged sweat rate >650 g/h, steep increase |
| + 38 to + 46 | very strong heat stress | – core to skin temperature gradient < 1K (at 30 min)  – increase in rectal temperature at 30 min |
| + 32 to + 38 | strong heat stress | – dynamic Thermal Sensation at 120 min >+2  – averaged sweat rate > 200 g/h  – increase in rectal temperature at 120 min  – latent heat loss >40 W at 30 min  – instantaneous change in skin temperature > 0 K/min |
| + 26 to + 32 | moderate heat stress | – change of slopes in sweat rate, rectal temperature and skin temperature  – occurrence of sweating at 30 min  – steep increase in skin wettedness |

**Table S3** List of International Classification of Diseases codes mapped to infertility in the GBD 2021.

| **Cause Name** | **ICD 10** | **ICD 9** |
| --- | --- | --- |
| Male infertility | N46-N46.02, N46.022-N46.12, N46.122-N46.9 | 606-606.9, V26.5, V26.52 |
| Female infertility | N97-N98.9 | 628-628.9,V26-V26.49,V26.51, V26.8-V26.9, V59.7-V59.74 |

**Table S4** Multicollinearity test for variables.

| **Variables** | **VIF** |
| --- | --- |
| *Multicollinearity test for UTCI≥26 ℃, SDI, PM, NO_2_, and O_3_.* | |
| Universal Thermal Climate Index≥26 ℃ | 2.05 |
| Sociodemographic index | 2.77 |
| Particulate matter | 1.91 |
| Nitrogen dioxide | 2.5 |
| Ozone | 1.75 |
| *Multicollinearity test for heat stress levels, SDI, PM, NO_2_, and O_3_.* | |
| Moderate heat stress | 1.56 |
| Strong heat stress | 1.65 |
| Very strong heat stress | 2.47 |
| Extreme heat stress | 1.61 |
| Sociodemographic index | 2.91 |
| Particulate matter | 2.64 |
| Nitrogen dioxide | 2.58 |
| Ozone | 1.80 |

VIF, variance inflation factor.

As commonly used rules of thumb, VIF values <5 generally indicate no substantial multicollinearity, values of 5–10 warrant closer examination, and values >10 may indicate serious multicollinearity; however [1,2].

References:

[1] O’Brien RM. A caution regarding rules of thumb for variance inflation factors. Qual Quant. 2007;41:673–690. doi:10.1007/s11135-006-9018-6
[2] Vatcheva KP, Lee M, McCormick JB, Rahbar MH. Multicollinearity in regression analyses conducted in epidemiologic studies. Epidemiology (Sunnyvale). 2016;6(2):227. doi:10.4172/2161-1165.1000227

**Table S5** Spatial association between the average number of days with UTCI ≥ 26 °C and mean

male and female infertility prevalence under different Jenks classifications, 1990–2021.

| **Variable** | **Male** | | | **Female** | | |
| --- | --- | --- | --- | --- | --- | --- |
|  | Jenks-4 | Jenks-5 | Jenks-6 | Jenks-4 | Jenks-5 | Jenks-6 |
| Universal Thermal  Climate Index≥26 ℃ | 0.100** | 0.126** | 0.128** | 0.094** | 0.099* | 0.095* |
| Continent | 0.102* | 0.102* | 0.102* | 0.044 | 0.044 | 0.044 |
| Particulate matter | 0.100* | 0.101 | 0.131 | 0.059* | 0.039 | 0.063 |
| Sociodemographic index | 0.064 | 0.076 | 0.061 | 0.024 | 0.037 | 0.038 |
| Nitrogen dioxide | 0.050 | 0.070* | 0.092* | 0.056* | 0.070* | 0.094* |
| Ozone | 0.019 | 0.025 | 0.031 | 0.025 | 0.040 | 0.039 |

All values were calculated as the q value.

*P < 0.05, **P<0.001.

**Table S6** The characteristics of heat stress by SDI, continent, and latitude,1990-2021

| **Variables** | **Moderate heat stress**  **(day)** | **Strong heat stress**  **(day)** | **Very strong heat stress**  **(day)** | **Extreme heat stress**  **(day)** |
| --- | --- | --- | --- | --- |
| Global | 80.72 (62.99) | 108.85 (88.91) | 43.45 (55.68) | 6.82 (20.76) |
| **SDI** |  |  |  |  |
| Low | 88.10 (65.47) | 153.56 (71.64) | 77.01 (70.11) | 8.32 (18.62) |
| Low-middle | 93.84 (68.40) | 136.90 (92.27) | 38.85 (40.00) | 4.53 (14.31) |
| Middle | 89.61 (61.94) | 93.99 (85.05) | 27.38 (35.35) | 5.74 (19.61) |
| High-middle | 63.68 (48.69) | 47.23 (55.64) | 20.92 (37.13) | 8.50 (27.95) |
| High | 36.13 (26.44) | 14.60 (21.21) | 7.45 (22.60) | 6.62 (27.23) |
| **Continent** |  |  |  |  |
| Asia | 68.43 (29.45) | 101.15 (80.30) | 50.26 (42.90) | 15.14 (32.71) |
| Europe | 45.51 (26.15) | 16.80 (16.72) | 1.77 (3.36) | 0.00 (0.05) |
| Africa | 84.76 (65.23) | 146.74 (73.79) | 82.63 (70.86) | 9.56 (19.86) |
| North America | 142.32 (89.12) | 152.65 (89.49) | 18.66 (21.77) | 0.08 (0.26) |
| South America | 65.68 (22.84) | 170.86 (93.00) | 34.14 (27.77) | 0.07 (0.22) |
| Oceania | 152.98 (88.87) | 123.42 (80.03) | 15.10 (27.32) | 1.48 (4.23) |
| **Latitude** |  |  |  |  |
| low | 97.69 (72.35) | 157.96 (77.35) | 62.50 (61.08) | 9.70 (25.02) |
| middle | 57.16 (22.77) | 31.24 (25.06) | 13.49 (23.19) | 2.33 (9.10) |
| high | 10.87 (7.79) | 1.09 (1.61) | 0.05 (0.16) | 0.00 (0.00) |

SDI, sociodemographic index

**Table S7** Subgroup interaction analyses for heat stress score and infertility prevalence with FDR-adjusted P values

| **Subgroup** | **Male** | |  | **Female** | |
| --- | --- | --- | --- | --- | --- |
|  | P for interaction | FDR-adjusted P |  | P for interaction | FDR-adjusted P |
| SDI | <0.001 | <0.001 |  | <0.001 | <0.001 |
| Continent | 0.035 | 0.035 |  | 0.027 | 0.040 |
| Latitude | 0.007 | 0.010 |  | 0.255 | 0.255 |

Heat stress score (weighted scores of very strong and extreme heat stress).

FDR, False Discovery Rate.

**Table S8** Association between male and female infertility ASPR and heat stress levels (Sensitivity analysis 1)

| **Heat stress level** | **Male** | | **Female** | |
| --- | --- | --- | --- | --- |
|  | β (95% CI) | P value | β (95% CI) | P value |
| Moderate heat stress | 0.526 (-0.211, 1.263) | 0.155 | 0.616 (-1.220, 2.452) | 0.499 |
| Strong heat stress | -0.000 (-0.585, 0.585) | 1 | -1.129 (-2.626, 0.367) | 0.134 |
| Very strong heat stress | 0.585 (-0.434, 1.605) | 0.25 | 1.475 (-0.893, 3.843) | 0.213 |
| Extreme heat stress | 0.557 (-1.966, 3.080) | 0.656 | 7.139 (0.828, 13.450) | 0.028 |

Sensitivity analysis 1: Estimates were obtained from two-way fixed-effects models including country/region and year fixed effects, with Driscoll-Kraay standard errors to account for serial correlation and cross-sectional dependence.

Adjustments were made for SDI, PM, NO₂ and O₃.

**Table S9** Association between male and female infertility ASPR and heat stress levels (Sensitivity analyses 2–5)

| **Group** | **Male** | | **Female** | |
| --- | --- | --- | --- | --- |
|  | β (95% CI) | P value | β (95% CI) | P value |
| **Sensitivity analyses 2:Using log-transformed infertility prevalence** | | | | |
| Moderate heat stress | -0.000 (-0.000, 0.000) | 0.981 | -0.000 (-0.001, 0.001) | 0.989 |
| Strong heat stress | -0.000 (-0.001, 0.000) | 0.126 | -0.001(-0.002, -0.000) | 0.008 |
| Very strong heat stress | 0.001 (0.000, 0.001) | <0.001 | 0.002 (0.001, 0.003) | <0.001 |
| Extreme heat stress | 0.002 (0.001, 0.004) | 0.001 | 0.005 (0.003, 0.008) | <0.001 |
| **Sensitivity analyses 3: Considering country-level population density** | | | | |
| **Further adjusting country-level population density** | | | | |
| Moderate heat stress | 0.543 (0.012, 1.074) | 0.046 | 1.176 (0.003, 2.350) | 0.05 |
| Strong heat stress | 0.397 (-0.061, 0.854) | 0.091 | -0.076 (-1.072, 0.920) | 0.882 |
| Very strong heat stress | 1.303 (0.687, 1.919) | <0.001 | 2.220 (0.874, 3.566) | 0.001 |
| Extreme heat stress | 0.866 (-0.575, 2.311) | 0.238 | 6.182 (2.976, 9.387) | <0.001 |
| **Scenario 1: Countries with population density in the highest tertile (Q3) and land area in the lowest tertile (Q1)** | | | | |
| Moderate heat stress | 0.250 (-0.295, 0.795) | 0.375 | 0.949 (-0.431, 2.331) | 0.181 |
| Strong heat stress | -0.027 (-0.593, 0.538) | 0.926 | -0.224 (-1.651, 1.204) | 0.76 |
| Very strong heat stress | 0.413 (-0.535, 1.361) | 0.4 | 0.817 (-1.563, 3.201) | 0.508 |
| Extreme heat stress | 0.221 (-1.943, 2.385) | 0.844 | 0.782 (-4.651, 6.216) | 0.781 |
| **Scenario 2: Countries with population density in the lowest tertile (Q1) and land area in the highest tertile (Q3)** | | | | |
| Moderate heat stress | 1.733 (-0.447, 3.911) | 0.127 | 1.395 (-2.733, 5.523) | 0.516 |
| Strong heat stress | 1.149 (-0.411, 2.693) | 0.153 | 2.814 (-0.093, 5.702) | 0.062 |
| Very strong heat stress | 3.349 (1.676, 5.004) | <0.001 | 5.534 (2.442, 8.623) | <0.001 |
| Extreme heat stress | 1.232 (-1.581, 4.055) | 0.39 | 9.081 (3.817, 14.343) | <0.001 |
| **Sensitivity analyses 4: Further adjusting life expectancy, urbanization rate, and smoking prevalence (1990-2019)** | | | | |
| Moderate heat stress | 0.407 (-0.125, 0.938) | 0.134 | 1.174 (-0.003, 2.351) | 0.052 |
| Strong heat stress | 0.306 (-0.150, 0.762) | 0.19 | 0.023 (-0.974, 1.020) | 0.964 |
| Very strong heat stress | 0.971 (0.352, 1.590) | 0.002 | 1.593 (0.230, 2.955) | 0.023 |
| Extreme heat stress | 2.100 (0.642, 3.560) | 0.005 | 7.714 (4.448, 10.975) | <0.001 |
| **Sensitivity analyses 5: Long-term exposure by averaging exposures from the current year and prior 5 years (lag 0-5 year)** | | | | |
| Moderate heat stress | 0.240 (-0.680, 1.160) | 0.606 | 2.056 (0.086, 4.025) | 0.042 |
| Strong heat stress | 0.460 (-0.177, 1.098) | 0.16 | -0.423 (-1.808, 0.962) | 0.548 |
| Very strong heat stress | 2.102 (1.132, 3.073) | <0.001 | 3.393 (1.262, 5.516) | 0.002 |
| Extreme heat stress | 1.474 (-0.746, 3.699) | 0.196 | 8.107 (3.203, 12.989) | 0.001 |

Sensitivity analysis 2: Infertility prevalence data for 1990–2021 were log-transformed to approximate normality. Adjustments were made for SDI, PM, NO₂ and O₃.

Sensitivity analysis 3: Adjustments were made for SDI, PM, NO₂, O₃ and population density.

Sensitivity analysis 4: Adjustments were made for SDI, PM, NO₂, O₃, life expectancy, urbanization rate, and smoking prevalence (1990-2019).

Sensitivity analysis 5: Long-term exposure was assessed by averaging exposures from the current year and the preceding 5 years (lag 0–5 years). Adjustments were made for SDI, PM, NO₂ and O₃.

**Table S10** Interaction tests for differences in the associations between heat stress levels and infertility prevalence before and after 2010 among male and female.

| **Heat stress level** | **P for interaction** | |
| --- | --- | --- |
|  | Male | Female |
| Moderate heat stress | <0.001 | <0.001 |
| Strong heat stress | 0.742 | 0.562 |
| Very strong heat stress | <0.001 | 0.081 |
| Extreme heat stress | 0.364 | 0.839 |

P for interaction tests whether the association between each heat stress level and infertility prevalence differed between 1990–2009 and 2010–2021.

**Figure S1** Data screening process for the present study

**Figure S2** The change in number of moderate heat stress, strong heat stress, very strong heat stress and extreme heat stress days from 1990 to 2021.

LOESS, locally estimated scatterplot smoothing.

**Figure S3** Directed Acyclic Graph showing the assumed associations between heat stress, covariates, and infertility.
